# Comprehensive analysis of cuproptosis-related long noncoding RNA immune infiltration and prediction of prognosis in patients with bladder cancer

**DOI:** 10.3389/fgene.2022.990326

**Published:** 2022-09-14

**Authors:** Yaoyu Zhang, Xiaodong Li, Xiaowei Li, Youguang Zhao, Tingting Zhou, Xin Jiang, Yang Wen, Wenjun Meng, Shadan Li

**Affiliations:** ^1^ Department of Urology, The Affiliated Hospital of Southwest Medical University, Luzhou, China; ^2^ Department of Urology, The General Hospital of Western Theater Command, Chengdu, China; ^3^ Department of Biotherapy, Cancer Center, West China Hospital, Sichuan University, Chengdu, China

**Keywords:** bladder cancer, lncRNA, cuproptosis, prognostic model, bioinformatics, immune status

## Abstract

**Background:** Bladder cancer (BCa), among the world’s most common malignant tumors in the urinary system, has a high morbidity and mortality. Though cuproptosis is a new type of cell death mediated by lipoylated tricarboxylic acid (TCA) cycle proteins, the role of cuproptosis-related long noncoding RNAs (crlncRNAs) in bladder tumors awaits further elucidation. In this paper, we tried to explore how important crlncRNAs are for BCa.

**Methods:** The crlncRNAs were first obtained through Pearson correlation analysis of the RNA-seq data and corresponding clinical data downloaded from The Cancer Genome Atlas (TCGA). Then, three lncRNAs were acquired by Cox regression and Lasso regression to build a prognostic model of crlncRNAs for verification. In the meantime, clinicopathological correlation analysis, Kyoto Encyclopedia of Genes and Genomes (KEGG) enrichment analysis, principal component analysis (PCA), immunoassay, and half-maximal inhibitory concentration prediction (IC50) were carried out. Then, an entire tumor was classified into two clusters by crlncRNA expression to further discuss the differences in prognosis, immune status and drug susceptibility among different subgroups.

**Results:** We obtained a total of 152 crlncRNAs and built a risk model for screened crlncRNAs. We validated the model and found that calibration charts feature a high consistency in verifying nomogram prediction. Receiver operating characteristic (ROC) curve and univariate and multivariate Cox regression suggested that this model can be applied as an independent prognostic factor of bladder cancer due to its high accuracy. According to KEGG analysis, high-risk groups were enriched in cancer and immune-related pathways. During tumor immunoassay, noticeable differences were observed in both immune infiltration and checkpoints between high- and low-risk patients. Of the two subgroups divided among patients by consensus clustering, cluster 2 had a better prognosis, whereas cluster 1 had higher immunoreactivity scores, more immune cell infiltrations and immune checkpoint expressions, and different sensitivities to drugs.

**Conclusion:** The research findings demonstrate that crlncRNAs can be used to predict the prognosis and immune microenvironment of patients suffering from BCa, and differentiate between BCa subgroups to improve the individual therapy of BCa.

## Introduction

As one of the world’s 10 most common cancers, bladder tumors have been diagnosed in more than 550,000 patients yearly (Richters et al., 2020). Among the 80% of the patients with non-muscle-invasive bladder cancer (NMIBC), postoperative recurrence occurs in approximately 50% of them after surgical treatment (Cambier et al., 2016). More recently, with further researches into the pathogenesis of bladder cancer (BCa), targeted therapy and immunotherapy have been increasingly applied to treat patients with advanced BCa (Patel et al., 2020). However, due to the lack of specific biomarkers, a proportion of incurably ill patients still cannot get effective treatment at an early stage (Tran et al., 2021). Therefore, new diagnostic strategies and individual-based treatment protocols are needed for BCa.

Copper is an indispensable coenzyme for essential enzymes involved in various biological processes (Kim et al., 2008). A recent report has demonstrated that patients with cancer have significantly elevated levels of copper in their serum and tumor tissues and that copper can directly bind to the lipoylated components of tricarboxylic acid (TCA) cycle. The build-up of copper-bound, lipoylated mitochondrial proteins and ensuing Fe-S cluster protein losses provoke proteotoxic stress and lead to a unique form of cell death. This novel form of cell death is called cuproptosis (Ishida et al., 2013; Li et al., 2022; Tsvetkov et al., 2022). Studies have reported that vitamin K2 remarkably accelerated the glucose breakdown process of BCa cells by up-regulating glucose consumption and lactate generation but held back the TCA cycle by lessening the content of Acetyl-CoA (Duan et al., 2020). This also suggests that cuproptosis may be a potential immunotherapeutic target for BCa.

Long noncoding RNAs (lncRNAs) are RNAs comprising a group of more than 200 nucleotides without protein-coding functions (Quinn and Chang, 2016) that affect every aspect of tumor cells by participating in mRNA expression and gene regulation (Huang et al., 2018), including tumorigenesis, tumor cell proliferation, and tumor metastasis (Mattick and Makunin, 2006; Cheetham et al., 2013). LncRNATTN-AS1, for instance, advances melanoma progression and metastasis by maintaining TTN expression (Wang et al., 2020); MALAT1 is important in the diagnosis and prognosis of multiple tumors (Goyal et al., 2021). However, studies on cuproptosis-related lncRNAs (crlncRNAs) in the prognosis of bladder tumors and tumor immune microenvironment (TME) have so far not been reported.

In this paper, we created a potential model on top of crlncRNAs (Zhang et al., 2022), which has a clinical application value for predicting the prognosis of BCa patients and selecting potential drugs.

## Materials and methods

### Access to the information of patients with Bladder cancer

We downloaded the RNA-seq transcription data and clinical information of BCa patients from The Cancer Genome Atlas (TCGA) database (https://tcga-data.nci.nih.gov/tcga/). To reduce statistical bias, we ruled out the patients with missing overall survival (OS) or OS (<30 days), and kept the RNA-seq data and corresponding clinical information of 411 bladder tumor samples and 19 normal tissue samples.

### Selection and differential expression analysis of CrlncRNAs

Ten cuproptosis-relate genes (CRG) were gathered from previous studies and document retrieval (Supplementary Table S1) (Tsvetkov et al., 2022). The correlation of the 10 CRGs with lncRNA expression was measured with Pearson’s correlation coefficient. All crlncRNAs (152 in total) met the correlation coefficient standard (|PearsonR|) >0.3 and *p* < 0.001. Then, we obtained 60 differentially expressed lncRNAs [(Log2 fold change (FC) > 1, fdrFilter (FDR) < 0.05]after screening the synthetic data matrix by Strawberry Perl V-5.30.0 (https://www.perl.org/) and R software V-4.1.2 (https://www.rproject.org/) with “limma” R package.

### Creation and verification of risk signature

All the BCa datasets from the TCGA database were screened for lncRNAs related to survival from crlncRNA (*p* < 0.05), we performed least absolute shrinkage and selection operator (lasso) Cox analysis (Bunea et al., 2011) to screen out optimal 3 lncRNAs associated with BCa prognosis and use it to create the model. Risk score formula per patient: risk score = Ʃ[Exp (lncRNA) × coef (lncRNA)]. Then, we randomized all samples into Train and Test groups in a 1:1 ratio, classified BCa patients into high- and low-risk groups (Zhao et al., 2021) with the median risk score derived from the above formula, and verified the correlation between clinical characteristics and risk groups by means of Chi-square tests to evaluate the prognostic value of the model constructed. The obtained receiver operating characteristic (ROC) curve and area under the ROC curve (AUC) were employed to measure the accuracy of the model, and all analyses were based on the “survival,” “caret,” “glmnet,” “rms,” “survminer,” and “timeROC,” R package.

### Construction and calibration of predictive nomogram

A nomogram or alignment diagram was drawn by the ages and clinicopathological factors of risk groups, aiming to estimate the predictive effect of the risk scores obtained for 1-, 3-, and 5-years OS, and calibration curves were fitted to illustrate the predictive power of the established nomogram model. All analyses were based on the “rms” R package.

### Functional enrichment analysis

Pursuant to the median risk score, BCa patients fell into high- and low-risk groups, in which differentially expressed Kyoto Encyclopedia of Genes and Genomes (KEGG) pathways were searched for by gene set enrichment analysis (GSEA) software as per the criteria of *p* < 0.05.

### Investigation of umor immune microenvironment and immune checkpoints

To analyze the immune-cell factors in risk groups, we calculated the immune infiltration statuses among BCa patients from TCGA using TIMER, CIBERSORT, XCELL, QUANTISEQ, MCPcounter, EPIC, and CIBERSORT on TIMER2.0 (http://timer.cistrome.org/). At the same time, we downloaded the profile of infiltration estimation for all TCGA tumors on the same website. We used Wilcoxon signed-rank test, “limma,” “scales,” “ggplot2,” and “ggtext” R packages to examine the differences in immune infiltrating cell contents, and the results are given in the bubble chart. Also, we compared the high- and low-risk groups with respect to TME scores and activation of immune checkpoints through the “ggpubr” R package.

### Investigation of the model in clinical treatment

By using the “pRophetic” package and analyzing the expression matrix of BCa patients, we predicted the IC50 of BCa patients and eventually attained a handful of drug candidates associated with this model, which might be a therapy for BCa.

### consensus clustering

To probe into the response of BCa to immunotherapy, we divided the patients into clusters as per the expression of crlncRNAs. “ConsensusClusterPlus” (CC) R package were used in search of potential molecular subgroups, and principal component analysis (PCA), T distributed stochastic neighbor embedding (t-SNE), and Kaplan-Meier survival were made by the “Rtsne” R package. At the same time, we performed immunoassays and compared the prognosis and drug susceptibility of different subgroups with the help of “GSVA” Base and “pRRophetic” package.

### Statistical analysis

For statistical analysis and outcome display, R software (version 4.1.2) was utilized. Kaplan-Meier survival curves and log-rank analysis were applied to assess the differences in survival time between subgroups. Besides, we applied time-dependent ROC curve analysis via “survivalROC” R package to evaluate the predictive performance of the risk model. The independent prognostic value of the risk signature was confirmed by univariate and multivariate Cox regression. Subgroups based on different clinical were investigated for comprehensive assessment of the stability of risk signature. The differences between subgroups were explored by utilizing Student’s t-test and Wilcoxon signed rank test. A *p* < 0.05 indicated statistically significant for all analyses.

## Results

### Identification of crIncRNA

**FIGURE 1:**
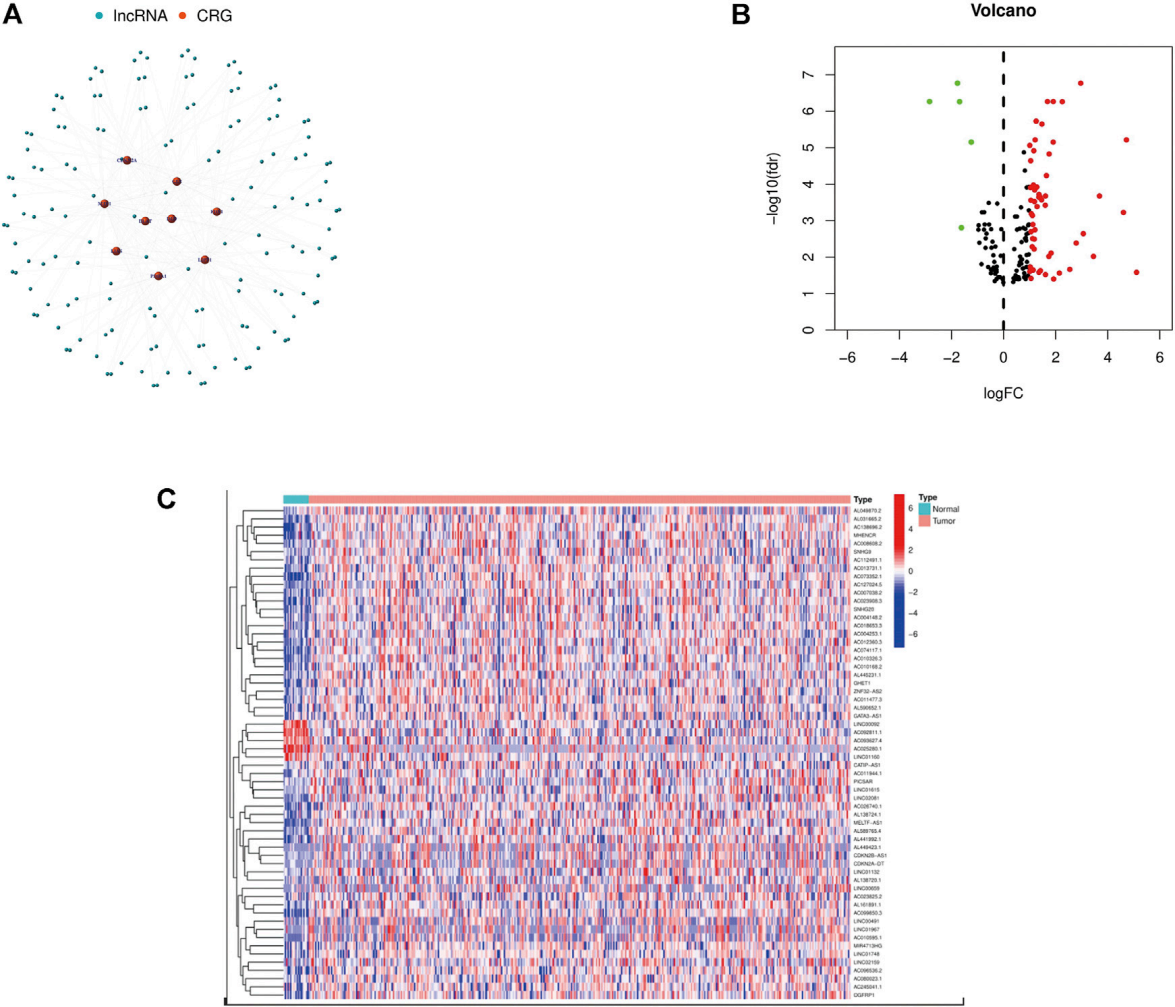
Identification of crlncRNAs in patients with BCa. **(A)** The network between CRGs and crlncRNAs (correlation coefficients >0.3 and *p* < 0.001). **(B)** A volcano map of differentially expressed CRG. **(C)** Heatmap of differentially expressed crlncRNAs.

We found 152 lncRNAs that had a co-expression relationship with CRG in BCa, and also made a network graph of co-expression relationship between CRG and crlncRNAs (Figure 1A; Supplementary Table S2). According to the expression of CRG and differentially expressed lncRNAs (|Log2FC|>1 and *p* < 0.05), we finally got 60 crlncRNAs, 55 genes were up-regulated and five genes down-regulated in expression (Figure 1B), and the most differentially expressed lncRNAs were selected for drawing a heat map (Figure 1C).

### Prognostic model construction and evaluation

**FIGURE 2:**
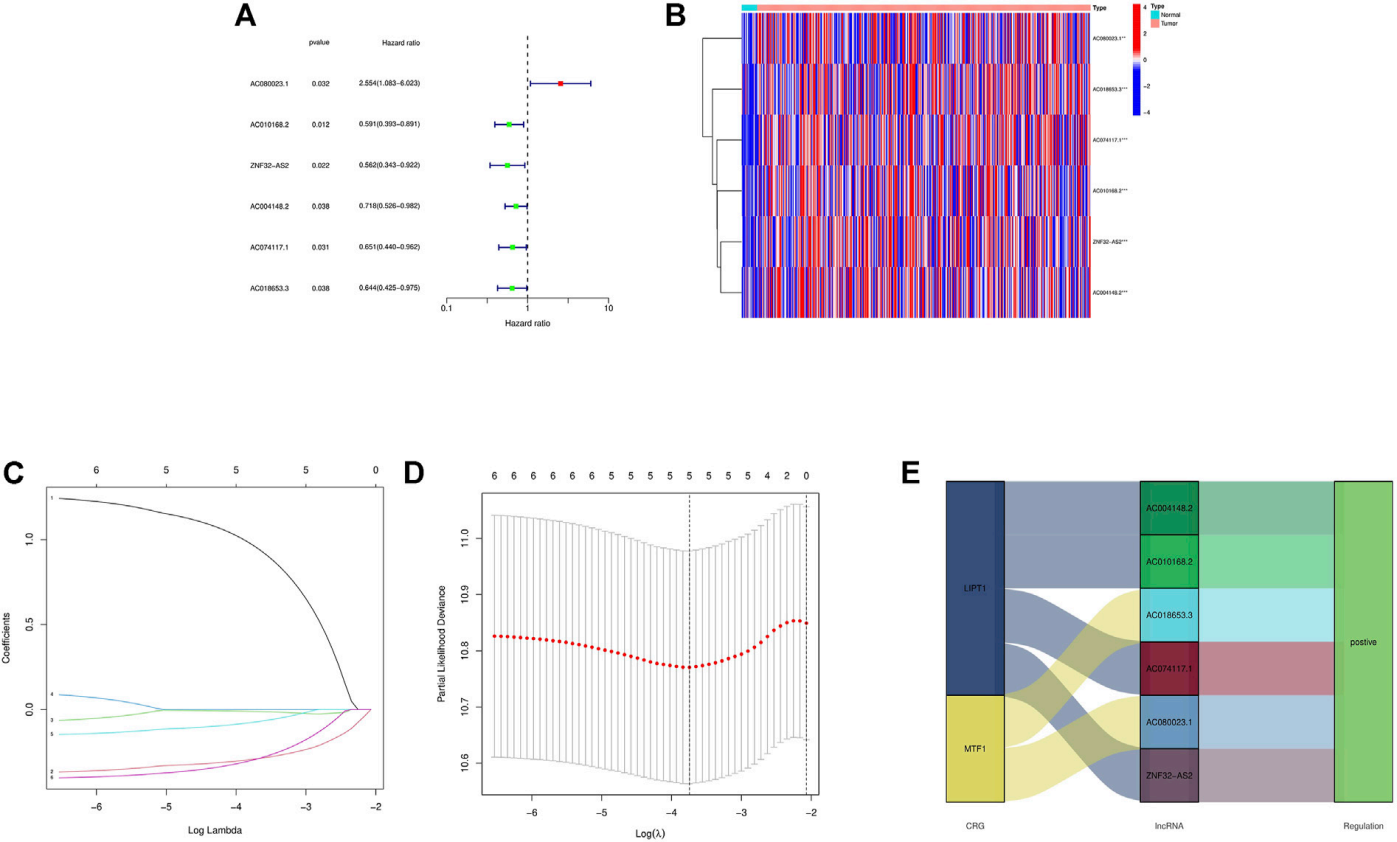
Extraction of crlncRNAs prognostic signature in BCa. **(A)** The prognostic lncRNAs extracted by univariate Cox regression analysis. **(B)** The expression profiles of 6 prognostic lncRNAs. **(C)** The 10-fold cross-validation for variable selection in the LASSO model. **(D)** The LASSO coefficient profile of crlncRNAs. **(E)** Sankey diagram of CRGs and crlncRNAs. ***p < 0.001, **p < 0.01, *p < 0.05.

**FIGURE 3:**
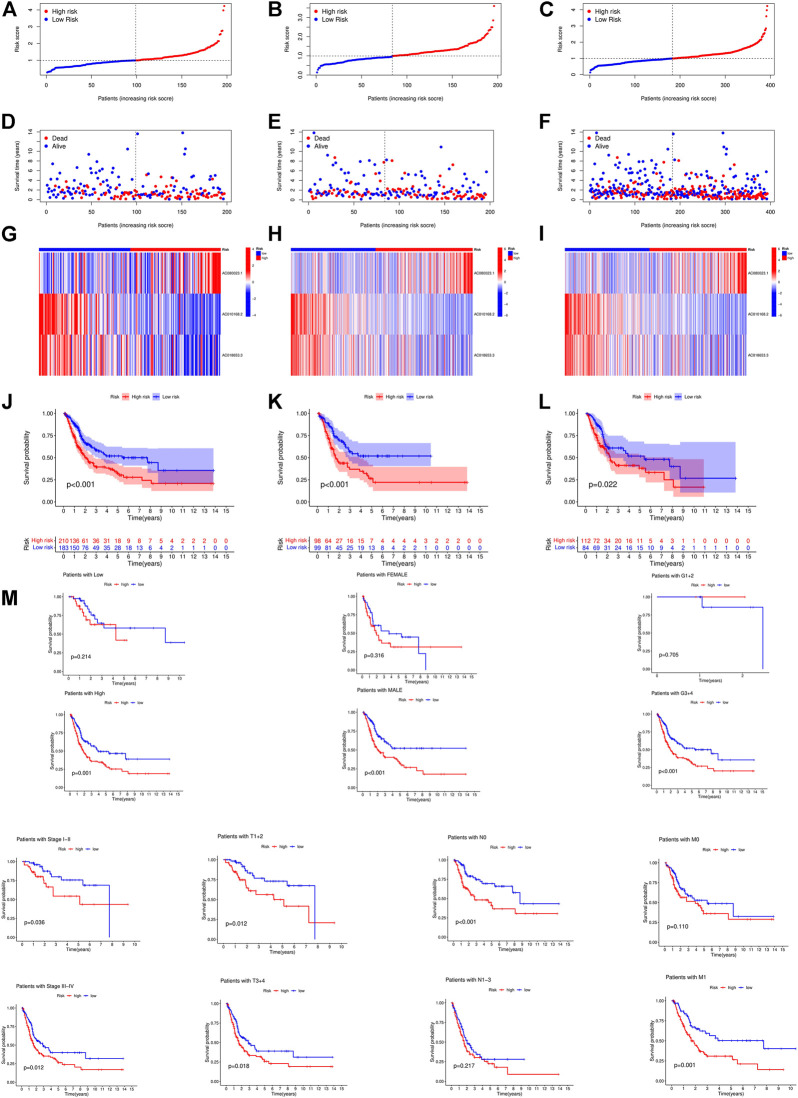
Prognosis value of the 3 crlncRNAs model in the train, test, and entire sets. **(A–C)** Exhibition of crlncRNAs model based on risk score of the train, test, and entire sets, respectively. **(D–F)** Survival time and survival status between low and high-risk groups in the train, test, and entire sets, respectively. **(G–I)** The heat map of 3 lncRNAs expression in the train, test, and entire sets, respectively. **(J–L)** Kaplan–Meier survival curves of OS (survival probability) of patients between low- and high-risk groups in the train, test, and entire sets, respectively. **(M)** Kaplan-Meier survival curves of OS (survival probability) prognostic value stratified by age, gender, grade, stage, T, N, or **(M)** between low- and high-risk groups in the entire set.

**FIGURE 4:**
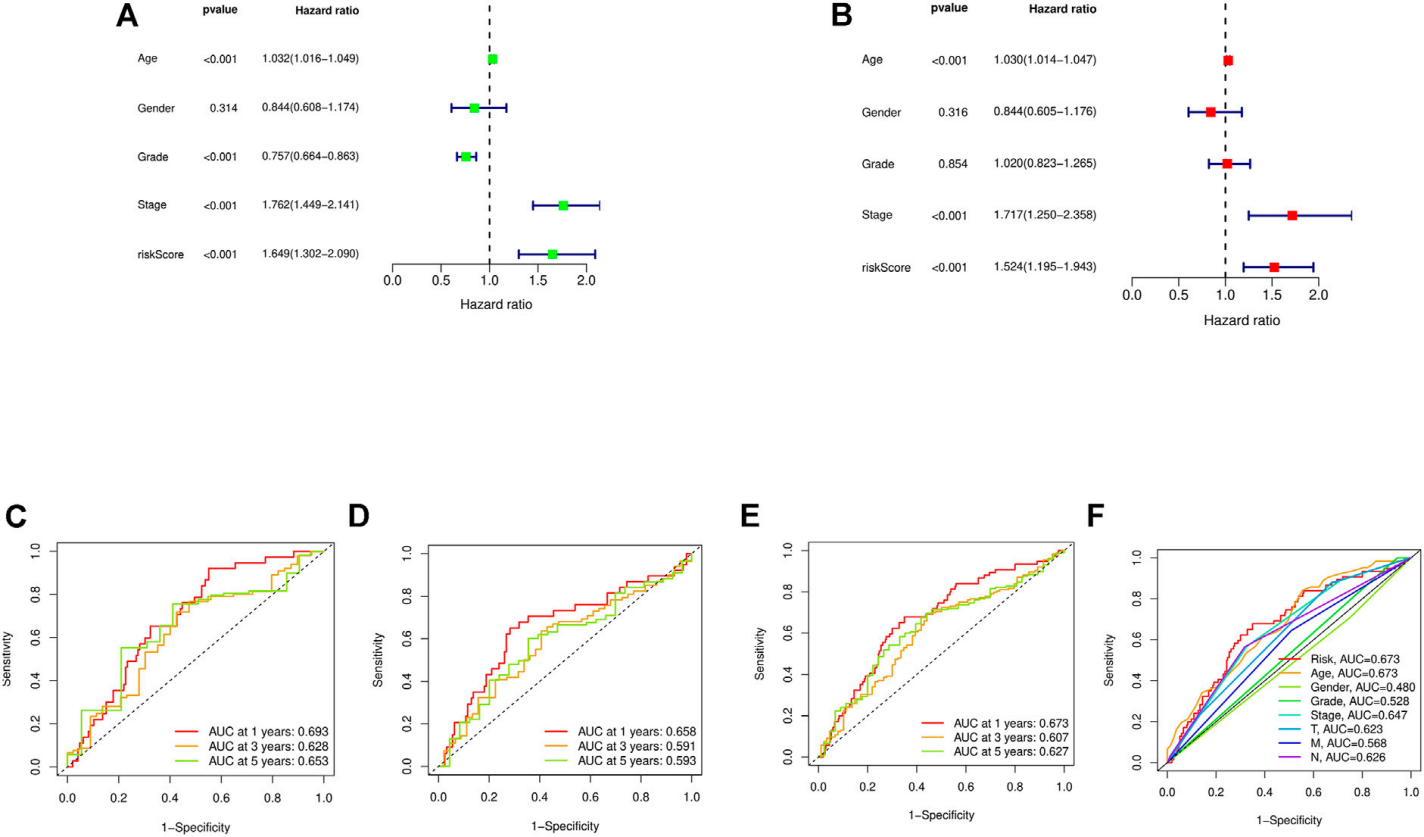
Assessment of the risk model. **(A,B)** Uni- and multi-Cox analyses of clinical factors and risk score with OS. **(C–E)** The 1-, 3-, and 5-years ROC curves of the train, test, and entire sets, respectively. **(F)** The ROC curves of risk score and clinical characteristics.

First, a forest plot and a heat map were made with the lncRNAs extracted by univariate Cox regression (Figures 2A,B), then three of the lncRNAs were verified by Lasso regression (Figures 2C,D) to build this model. In addition, the Sankey diagram indicated that the expression of all six lncRNAs were up-regulated (Figure 2E). The risk score formula adopted is like this: Risk score = AC080023.1 × (1.23466619462196) + AC010168.2 × (-0.413447164489597) + AC018653.3 × (-0.419348813769036) (Zhao et al., 2021). With this formula, we compared the risk score distribution, survival status, and survival time expression results of the patients were compared between low- and high-risk groups in the train, test, and entire sets, with the results showing that the high-risk group had a much shorter OS (Figures 3A–L). Clinicopathological characteristics showed the same results (Figure 3M). To verify whether the model is an independent prognosis predictor, univariate and multivariate Cox regression was implemented. The hazard ratio (HR) and 95% confidence interval (CI) of risk scores were 1.649 and 1.302-2.090 (*p* < 0.001) respectively in the univariate Cox regression, and 1.524 and 1.195-1.943 (*p* < 0.001) respectively in the multivariate Cox regression (Figures 4A,B). Also, two other independent prognostic parameters were discovered: age (1.030 and 1.014-1.047; *p* < 0.001) and stage (1.717 and 1.250-2.358; *p* < 0.001) (Figure 4B). Moreover, the susceptibility and specificity of the model to prognosis was evaluated using time-dependent ROC. The AUC were respectively 0.673, 0.607 and 0.627 for 1-, 3- and 5-years OS (Figures 4C–E), and the risk score AUC of the model was 0.673, revealing a stronger predictive power than other clinicopathological characteristics (Figure 4F).

### Nomogram construction

**FIGURE 5:**
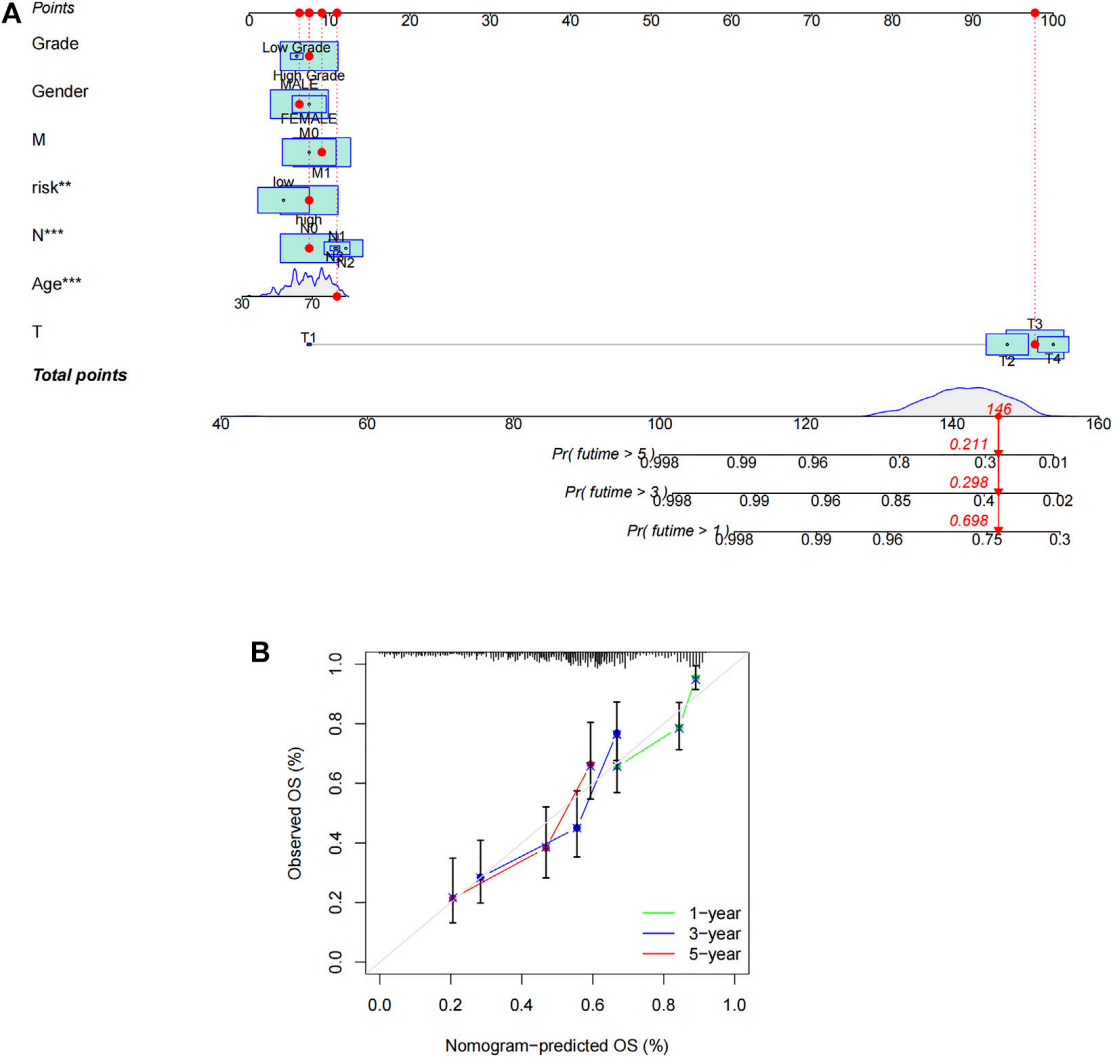
Construction of nomogram. **(A)** Nomogram for predicting overall survival. **(B)** The calibration curves for 1-, 3-, and 5-years OS.

Based on the risk score, age, gender, and other clinical features described above, we drew an alignment diagram projecting 1-, 3-, and 5-years OS and OS incidence in patients diagnosed with bladder tumors (Figure 5A), and, used the 1-, 3-, and 5-years calibration plots to verify whether the nomogram well tallied with prediction. The results proved that it coincided with the actual effect (Figure 5B). Thus, risk characteristics are clearly associated with the development of BCa and might be a valuable tool for the clinical management of patients.

### Functional enrichment analysis

**FIGURE 6:**
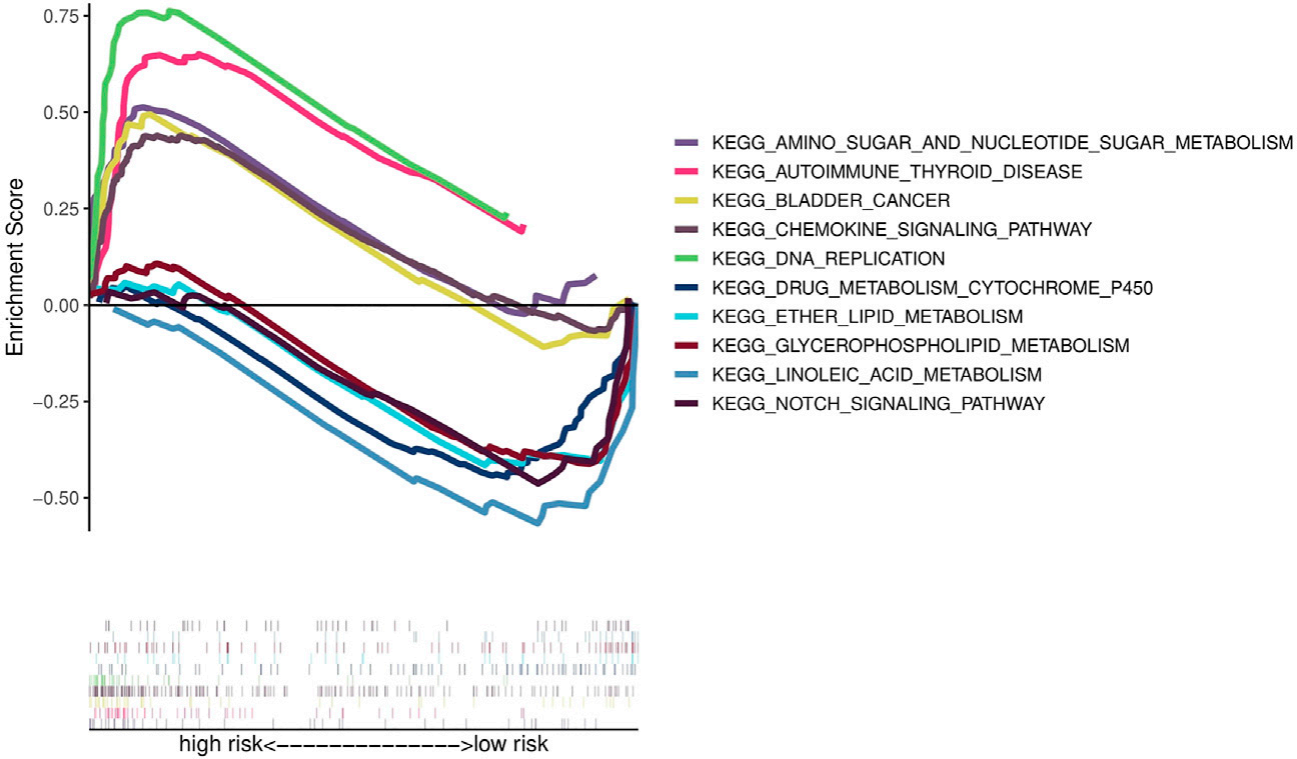
Results of KEGG analyses.

To investigate the functional differences in gene expression between high- and low-risk groups, we carried out functional enrichment analysis using GSEA software. The analysis indicates that the high-risk group is closely associated with the pathways in BCa, cell proliferation, nucleotide metabolism, inflammatory immune response and immune cell (Figure 6). Therefore, we conducted immune correlation analysis based on this risk model.

### Exploration of immune characteristics and clinical treatment in risk groups

**FIGURE 7:**
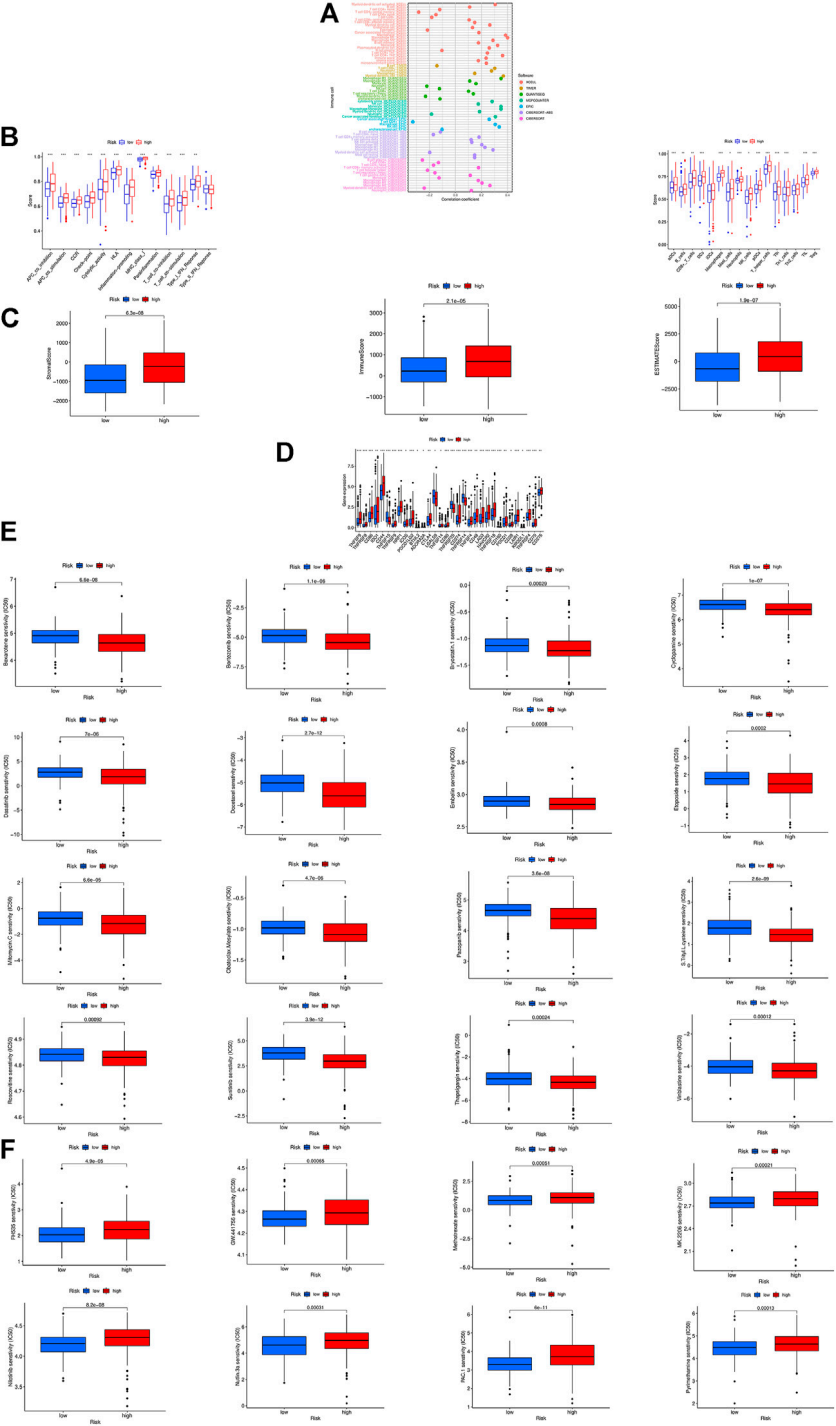
The investigation of tumor immune factors and immunotherapy. **(A)**The immune cell bubble of risk groups. **(B)** Immune-related pathways and immune cell in the high-risk group and the low-risk group are displayed in boxplots. **(C)** The comparison of immune-related scores between low- and high-risk groups. **(D)** The difference of 32 checkpoints expression in risk groups. **(E,F)** Sensitivity to different types of chemotherapeutic agents. ***p < 0.001, **p < 0.01, *p < 0.05.

In the bubble chart (Figure 7A; Supplementary Table S3), immune cells are more relevant to the high-risk groups on whatever platforms, such as T cell CD8^+^ effector memory, Cancer associated fibroblast, Macrophage M1 at XCELL, T cell CD8^+^, and Neutrophil at TIMER, Myeloid dendritic cell, Cancer associated fibroblast, NK cell at MCPCOUNTER, and Macrophage at EPIC. NK cells and their cytotoxicity figure prominently in immunity and cancer according to researches (Huntington et al., 2020). ssGSEA analysis reveals that the proportion of almost all immune cell subpopulations as well as the component levels and functions of relevant pathways are lower in the low-risk group compared to the high-risk group (Figure 7B), and the high-risk group has a higher immune score and ESTIMAT (microenvironment) score (Figure 7C). Most immune checkpoints are more active in the high-risk group (Figure 7D). All this indicates differences in immune status between high- and low-risk groups, providing relevant basis for development of bladder tumor immunotherapies. In addition, the research on the susceptibility of different patient groups to antitumor drugs suggests that high-risk groups are more sensitive to some drugs like Bortezomib, Bexarotene, Dasatinib and Sunitinib (Figure 7E), whereas low-risk groups more sensitive to Pyrimethamine, Methotrexate, and Nilotinib (Figure 7F). It is instructive to the selection of appropriate drugs by risk group.

### Prognosis and immunotherapy prospect of different BCa subgroups

**FIGURE 8:**
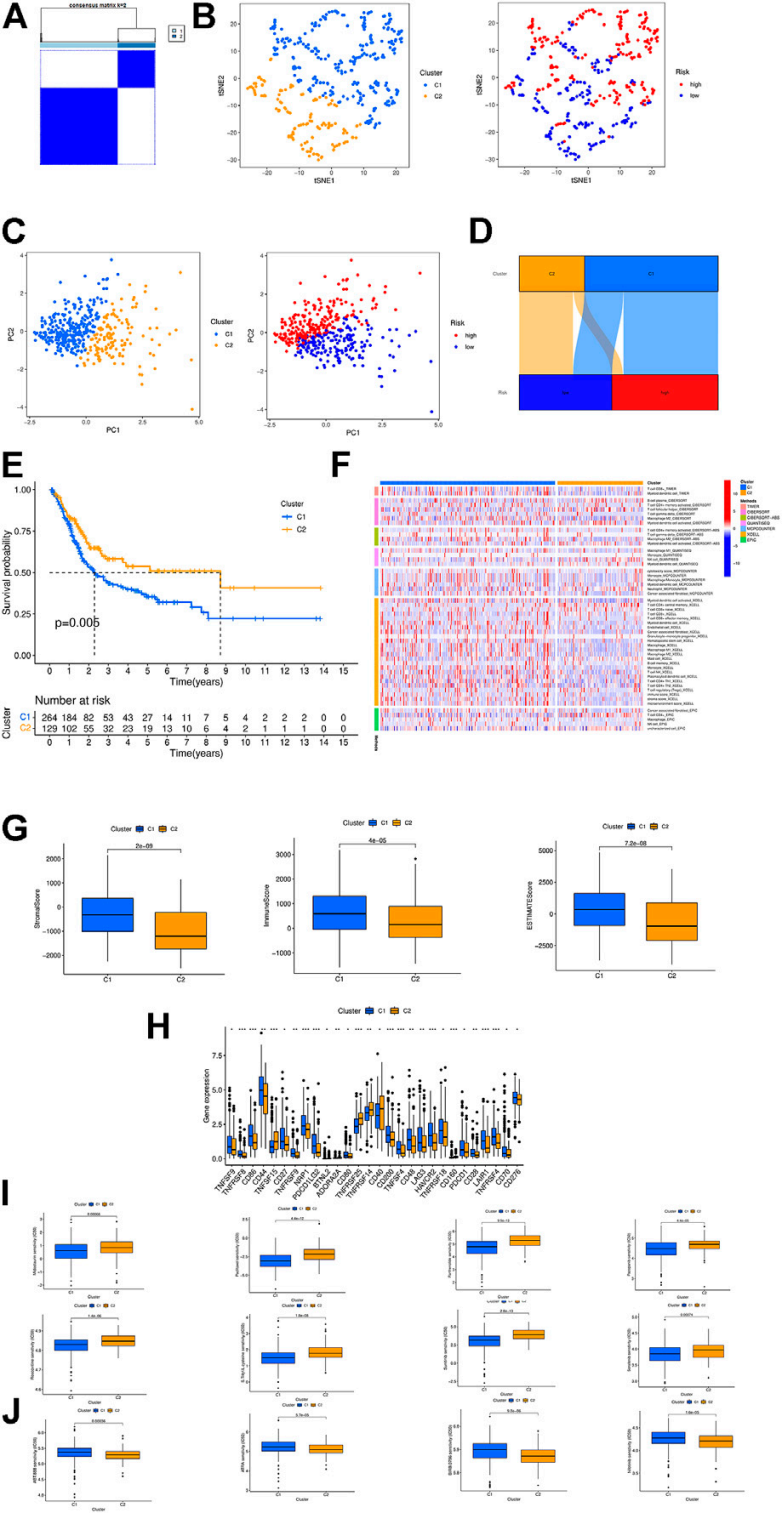
Consensus Clustering based on prognostic crlncRNAs in BC. **(A)** Patients divided into two clusters by ConsensusClusterPlus. **(B)** The t-SNE of two clusters. **(C)** The PCA of risk groups and clusters. **(D)** The Sankey diagram of different clusters. **(E)** The Kaplan-Meier survival curves of OS in clusters. **(F)** The heat map of immune cells in clusters. **(G)** The comparison of immune-related scores between clusters 1 and 2. **(H)** The difference of 28 checkpoints expression in clusters. **(I,J)** Sensitivity to different clusters of chemotherapeutic agents. ***p < 0.001, **p < 0.01, *p < 0.05.

According to the expression of crlncRNAs screened for prognostic evaluation, the patients were again grouped into two clusters by CC R package (Figure 8A; Supplementary Table S1) (Wilkerson and Hayes, 2010), and the t-SNE results could more explicitly show the distribution of the two clusters and high- and low-risk groups (Figure 8B). PCA indicated differences in both risk groups and clusters (Figure 8C). In the Sankey diagram, it can be seen that most patients in cluster 1 are in the high-risk group while most patients in cluster 2 are in the low-risk group (Figure 8D). Subsequently, we performed survival analysis to explore whether there is an obvious difference in the prognosis of patients between subtypes. The results show that the survival time is much longer in cluster 2 than in cluster 1 (Figure 8E). According to the analysis conducted via different platforms, the degree of immune cell infiltration is higher in cluster 1 (Figure 8F), which has significantly higher Immune (*p* < 0.0001), Stromal (*p* < 0.001) and ESTIMATE (*p* < 0.0001) scores than cluster 2 (Figure 8G), and most of the immune checkpoints also show a better activity in cluster 1 (Figure 8H). Based on the results above, we can argue that cluster 1 and 2 may have different immunotherapeutic responses, and that cluster 1 is more effective for immunotherapy. Drug susceptibility comparison revealed immunotherapeutic drugs like Sunitinib, Sorafenib and Roscovitine had a higher sensitivity in cluster 1 (Figure 8I), and drugs such as Nilotinib had a higher sensitivity in cluster 2 (Figure 8J). For this reason, we might further investigate the immunotherapeutic response of BCa to boost precision medicine in the patients diagnosed with BCa.

## Discussion

BCa is a highly aggressive tumor with a unfavorable prognosis (Grayson, 2017). Treatment options for advanced BCa have expanded to, without limitation, immunotherapy with checkpoint inhibitors, targeted therapies, and antibody drug couples, but the effect remains poor (Lenis et al., 2020). So, research should be done on effective individualized treatment for patients with BCa. Cuproptosis is a novel mode of cell death distinct from apoptosis, ferroptosis, pyroptosis and necrosis (Tsvetkov et al., 2022), and reports have shown a new way of treating cancer by fully exerting the physiological and pathological roles of copper (Li et al., 2022). We therefore created a crlncRNAs signature to predict the prognosis of BCa so that we can help offer potential drug treatments.

During the present research, we investigated the role of crlncRNA in BCa and identified the lncRNAs with a prognostic value. By Cox regression and Lasso regression, we built a prognostic model consisting of three lncRNAs. The OS was quite lower in the high-risk group than in the low-risk group. The accuracy of the prognostic model was demonstrated by ROC curve and calibration curve. An alignment diagram was created to estimate the prognosis of BCa, and the results coincided with prediction. Clinicopathological analysis and survival analysis showed the model was highly sensitive to survival prediction. Furthermore, univariate and multivariate Cox analysis suggested that the model could be employed as an independent prognosis predictor.

According to GSEA analysis, the risk model was enriched in several immune-related pathways, e.g., pathways in BCa, cell proliferation, nucleotide metabolism, inflammatory immune response and immune cell. Not only that, we found higher expression levels of immune checkpoint-related genes in the high-risk group, further supporting the conjuncture that immune checkpoint inhibitor therapy is more effective in the high-risk group. We also investigated the relationship between the model and chemotherapeutic drug response to enable personalized treatment strategies. The above studies may present valuable references for future immunotherapies of BCa.

Molecular subtype, also known as cluster, is related to tumor immune suppression and microenvironment (Ajani et al., 2017; Smyth et al., 2020). Subtypes vary with immune and TME scores, which in turn lead to different prognosis and immunotherapeutic responses (Zeng et al., 2019; DeBerardinis, 2020). Therefore, we carried out consensus clustering analysis according to the expression of prognostic crlncRNAs and divided BCa patients into two clusters. In the PCA scatter diagram the patients in different clusters exhibited distinct intrinsic biological characteristics, and survival analysis showed that cluster 2 had a better prognosis, whereas cluster 1 outperformed it with respect to immune, mesenchyme and estimation scores. Immune infiltration and immune checkpoints suggest that in cluster 1 there is an increased number of highly infiltrated CD86 and LAG3 cells, which are more active in boosting inflammation, and that CD44 has a higher activity and is more able to promote tumor metastasis (Hassn Mesrati et al., 2021). Therefore, cluster 1 may be more effective for immunotherapy. Furthermore, the two clusters have different sensitivities to immunotherapeutic drugs, and can thus guide drug selection for subsequent treatment for BCa. All in all, crlncRNAs can serve as a guide to individual treatment, in addition to prognosis prediction.

The present study has some limitations and deficiencies. First, our investigation was based on the TCGA dataset rather than our own data, and the investigation results were not verified either *in vitro* or *in vivo*. The biological functions need further exploration. Second, the potential molecular mechanism of crlncRNA in BCa remains to be further validated experimentally. Third, we could not demonstrate the value of the prognostic model due to the lack of clinical follow-up data.

In conclusion, we developed a prognostic model based on crlncRNAs in BCa patients, which has been shown to have a high predictive accuracy. Because of its significant value in predicting immune cell infiltration, immune function, tumor microenvironment, drug sensitivity etc. In patients with BCa, the model can help improve the prognosis and individual treatment of BCa.

## Data Availability

Publicly available datasets were analyzed in this study. This data can be found here: http://portal.gdc.cancer.gov/repository.

## Supplementary material

SUPPLEMENTARY FIGURE S1 The heat map, cumulative distribution function (CDF) plot, and the consensus CDF plots of consensus clustering matrix.

SUPPLEMENTARY TABLE S1 The table of CRGs refers to previous studies and document retrieval.

SUPPLEMENTARY TABLE S2 The network data of CRGs and crlncRNAs.

SUPPLEMENTARY TABLE S3 The profile of significantly differently infiltrated immune cells between risk groups in different platforms.
