# Supplementary material for: Comprehensive analysis of cuproptosis-related long noncoding RNA immune infiltration and prediction of prognosis in patients with bladder cancer
Source: Front Genet. 2022 Sep 14;13:990326. doi: 10.3389/fgene.2022.990326 (PMC9515487; doi:10.3389/fgene.2022.990326)
Supplement: Supplementary file 1 [file Table1.DOCX]

| Cuproptosis-related genes |
| --- |
| \| FDX1 \| \| --- \| \| LIAS \| \| LIPT1 \| \| DLD \| \| DLAT \| \| PDHA1 \| \| PDHB \| \| MTF1 \| \| GLS \| \| CDKN2A \| |
