# Supplementary figures and images for: Comprehensive analysis of cuproptosis-related long noncoding RNA immune infiltration and prediction of prognosis in patients with bladder cancer
Source: Front Genet. 2022 Sep 14;13:990326. doi: 10.3389/fgene.2022.990326 (PMC9515487; doi:10.3389/fgene.2022.990326)

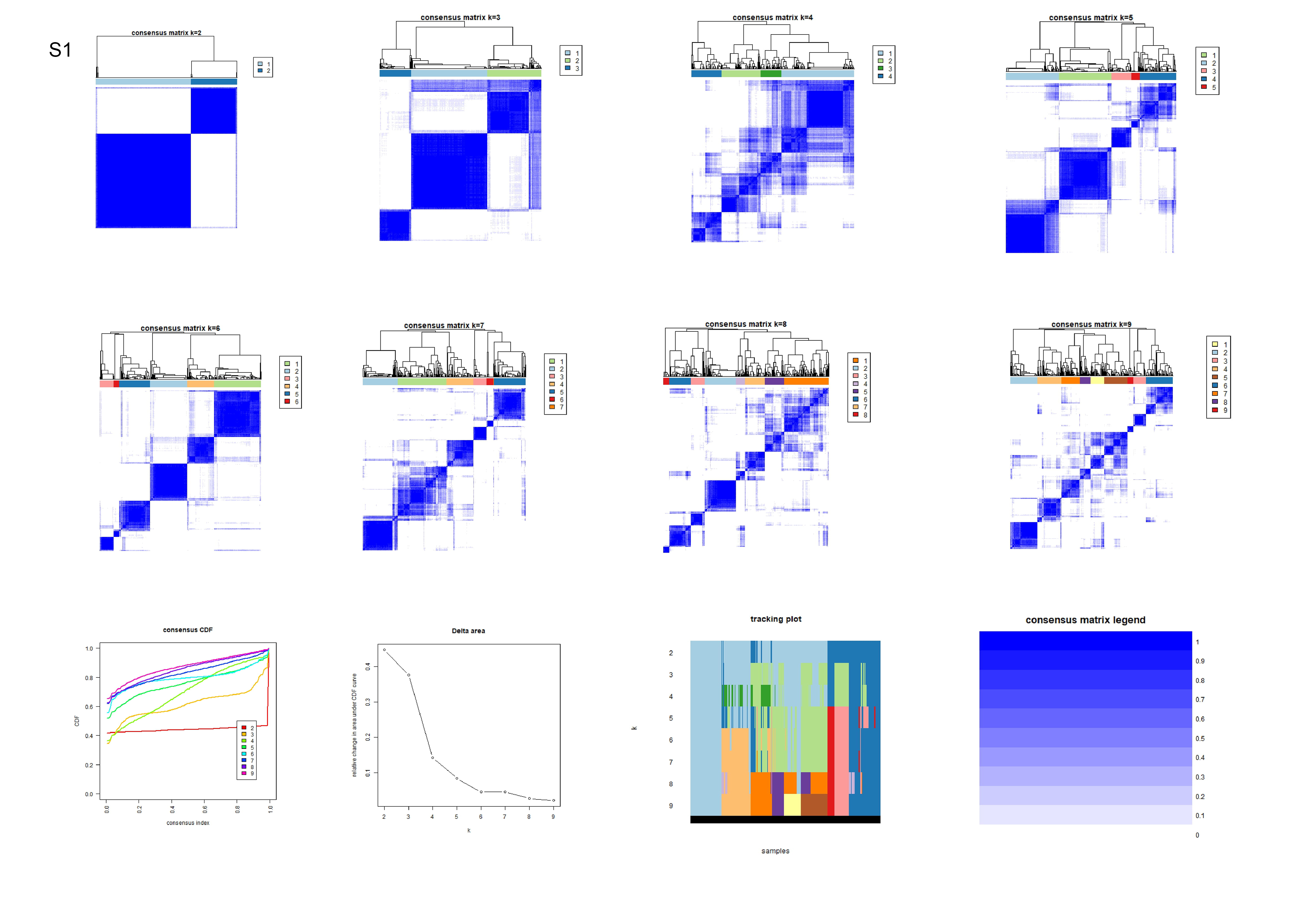

Supplement: Supplementary file 2 [file Image1.JPEG]
