# Supplementary material for: Comprehensive analysis of cuproptosis-related long noncoding RNA immune infiltration and prediction of prognosis in patients with bladder cancer
Source: Front Genet. 2022 Sep 14;13:990326. doi: 10.3389/fgene.2022.990326 (PMC9515487; doi:10.3389/fgene.2022.990326)
Supplement: Supplementary file 3 [file Table2.DOCX]

| CRG | lncRNA | cor | *p*-value | Regulation |
| --- | --- | --- | --- | --- |
| CDKN2A | AC011944.1 | 0.341669 | 0.00000000000107 | postive |
| MTF1 | AC024075.3 | 0.314584 | 0.00000000006820 | postive |
| MTF1 | RAB11B-AS1 | -0.32366 | 0.00000000001770 | negative |
| LIAS | AL133355.1 | 0.332013 | 0.00000000000494 | postive |
| MTF1 | AL450992.2 | 0.31708 | 0.00000000004730 | postive |
| PDHA1 | AC005225.2 | 0.308501 | 0.00000000016400 | postive |
| LIPT1 | AC021851.1 | 0.357139 | 0.00000000000008 | postive |
| FDX1 | AL445231.1 | 0.321631 | 0.00000000002410 | postive |
| GLS | RAP2C-AS1 | 0.318021 | 0.00000000004120 | postive |
| LIAS | IQCH-AS1 | 0.349602 | 0.00000000000029 | postive |
| MTF1 | AL031665.2 | -0.32902 | 0.00000000000784 | negative |
| GLS | AC011510.1 | 0.304546 | 0.00000000028700 | postive |
| LIPT1 | AP001462.1 | 0.311461 | 0.00000000010700 | postive |
| MTF1 | AC080023.1 | 0.323683 | 0.00000000001770 | postive |
| MTF1 | AC138696.2 | -0.30019 | 0.00000000052600 | negative |
| MTF1 | MIR4713HG | 0.33091 | 0.00000000000586 | postive |
| LIAS | LINC002481 | 0.347727 | 0.00000000000040 | postive |
| DLAT | AC061992.1 | 0.334134 | 0.00000000000355 | postive |
| PDHA1 | AC061992.1 | 0.60069 | 0.00000000000000 | postive |
| DLAT | LINC00092 | 0.374613 | 0.00000000000000 | postive |
| PDHA1 | LINC00092 | 0.619323 | 0.00000000000000 | postive |
| LIAS | AC108471.2 | 0.370418 | 0.00000000000001 | postive |
| CDKN2A | CDKN2A-DT | 0.808952 | 0.00000000000000 | postive |
| LIPT1 | LINC00910 | 0.324099 | 0.00000000001660 | postive |
| MTF1 | AC009041.4 | 0.320151 | 0.00000000003000 | postive |
| LIPT1 | CEBPA-DT | 0.316808 | 0.00000000004920 | postive |
| DLAT | AC090587.2 | 0.30638 | 0.00000000022200 | postive |
| MTF1 | AC090587.2 | 0.390104 | 0.00000000000000 | postive |
| LIPT1 | AC023908.3 | 0.379716 | 0.00000000000000 | postive |
| PDHA1 | CATIP-AS1 | 0.392203 | 0.00000000000000 | postive |
| LIPT1 | AC007038.2 | 0.304067 | 0.00000000030700 | postive |
| MTF1 | LINC01967 | 0.347128 | 0.00000000000044 | postive |
| LIPT1 | AL442128.2 | 0.318098 | 0.00000000004070 | postive |
| MTF1 | LINC02159 | 0.331839 | 0.00000000000507 | postive |
| MTF1 | LINC01748 | 0.323529 | 0.00000000001810 | postive |
| GLS | MAP3K20-AS1 | 0.327838 | 0.00000000000941 | postive |
| LIPT1 | AC009506.1 | 0.300627 | 0.00000000049500 | postive |
| LIPT1 | AC127024.5 | 0.319344 | 0.00000000003390 | postive |
| LIAS | AC019131.2 | 0.33213 | 0.00000000000485 | postive |
| LIPT1 | AC010168.2 | 0.305995 | 0.00000000023400 | postive |
| GLS | AC023825.2 | 0.350008 | 0.00000000000027 | postive |
| DLAT | AC099850.3 | 0.428923 | 0.00000000000000 | postive |
| MTF1 | AC099850.3 | 0.381259 | 0.00000000000000 | postive |
| DLD | AC002467.1 | 0.307662 | 0.00000000018500 | postive |
| CDKN2A | AL138724.1 | 0.311128 | 0.00000000011300 | postive |
| MTF1 | AL138762.1 | -0.35718 | 0.00000000000008 | negative |
| LIAS | GAS5 | 0.379924 | 0.00000000000000 | postive |
| LIAS | SRP14-AS1 | 0.306861 | 0.00000000020700 | postive |
| MTF1 | AC024075.1 | 0.409673 | 0.00000000000000 | postive |
| MTF1 | AC108449.2 | 0.360683 | 0.00000000000005 | postive |
| LIPT1 | AC005332.4 | 0.315972 | 0.00000000005570 | postive |
| MTF1 | LINC00630 | 0.354295 | 0.00000000000013 | postive |
| MTF1 | AC008608.2 | -0.35517 | 0.00000000000012 | negative |
| MTF1 | AC008764.2 | 0.376383 | 0.00000000000000 | postive |
| GLS | AC026356.1 | 0.36977 | 0.00000000000001 | postive |
| LIPT1 | ZNF32-AS2 | 0.334293 | 0.00000000000346 | postive |
| CDKN2A | AL441992.1 | 0.430591 | 0.00000000000000 | postive |
| LIPT1 | AL136295.2 | 0.330326 | 0.00000000000641 | postive |
| GLS | ATP1B3-AS1 | 0.304086 | 0.00000000030600 | postive |
| GLS | OSMR-AS1 | 0.369738 | 0.00000000000001 | postive |
| MTF1 | AC016888.1 | 0.300888 | 0.00000000047800 | postive |
| DLAT | AL161891.1 | 0.306865 | 0.00000000020700 | postive |
| LIPT1 | AC073352.1 | 0.306247 | 0.00000000022600 | postive |
| CDKN2A | PICSAR | 0.305221 | 0.00000000026100 | postive |
| MTF1 | CH17-340M24.3 | -0.37855 | 0.00000000000000 | negative |
| GLS | AC010503.4 | -0.30015 | 0.00000000052900 | negative |
| GLS | AL157394.1 | 0.301189 | 0.00000000045800 | postive |
| LIPT1 | GEMIN7-AS1 | 0.323757 | 0.00000000001750 | postive |
| CDKN2A | AL512598.1 | 0.340499 | 0.00000000000129 | postive |
| MTF1 | LINC02081 | 0.308015 | 0.00000000017600 | postive |
| DLD | LINC01160 | 0.319844 | 0.00000000003140 | postive |
| DLAT | AC093627.4 | 0.305059 | 0.00000000026700 | postive |
| PDHA1 | AC093627.4 | 0.303137 | 0.00000000034900 | postive |
| LIPT1 | STARD7-AS1 | 0.374209 | 0.00000000000000 | postive |
| LIAS | SCAMP1-AS1 | 0.334051 | 0.00000000000359 | postive |
| LIPT1 | AC004148.2 | 0.330311 | 0.00000000000643 | postive |
| LIPT1 | SNHG20 | 0.324069 | 0.00000000001670 | postive |
| MTF1 | AC026979.2 | -0.31129 | 0.00000000011000 | negative |
| GLS | SPINT1-AS1 | -0.34055 | 0.00000000000128 | negative |
| MTF1 | COX10-AS1 | 0.310266 | 0.00000000012700 | postive |
| LIPT1 | NIFK-AS1 | 0.301883 | 0.00000000041600 | postive |
| LIPT1 | FAM111A-DT | 0.302331 | 0.00000000039100 | postive |
| DLD | LINC00659 | 0.314831 | 0.00000000006580 | postive |
| DLAT | AL731577.2 | 0.33373 | 0.00000000000378 | postive |
| MTF1 | AL731577.2 | 0.445696 | 0.00000000000000 | postive |
| DLAT | AL139035.1 | 0.322913 | 0.00000000001990 | postive |
| PDHA1 | AC016924.1 | 0.399207 | 0.00000000000000 | postive |
| MTF1 | C1RL-AS1 | 0.319205 | 0.00000000003460 | postive |
| DLAT | AC005261.3 | -0.35581 | 0.00000000000010 | negative |
| MTF1 | AC010595.1 | 0.300655 | 0.00000000049300 | postive |
| MTF1 | AC245041.1 | 0.306223 | 0.00000000022700 | postive |
| MTF1 | SNHG9 | -0.35784 | 0.00000000000007 | negative |
| LIPT1 | GHET1 | 0.310498 | 0.00000000012300 | postive |
| PDHA1 | DM1-AS | 0.52749 | 0.00000000000000 | postive |
| LIPT1 | AC074117.1 | 0.322403 | 0.00000000002140 | postive |
| GLS | AC090559.1 | 0.375086 | 0.00000000000000 | postive |
| CDKN2A | LINC01132 | 0.333441 | 0.00000000000395 | postive |
| MTF1 | AL158212.3 | 0.343788 | 0.00000000000076 | postive |
| CDKN2A | AL589765.4 | 0.37013 | 0.00000000000001 | postive |
| GLS | AC025280.1 | 0.30631 | 0.00000000022400 | postive |
| CDKN2A | MELTF-AS1 | 0.308019 | 0.00000000017600 | postive |
| GLS | LINC-PINT | 0.352648 | 0.00000000000018 | postive |
| CDKN2A | EXOC3-AS1 | 0.351685 | 0.00000000000021 | postive |
| MTF1 | MHENCR | -0.31605 | 0.00000000005500 | negative |
| MTF1 | LINC00491 | 0.307866 | 0.00000000017900 | postive |
| CDKN2A | AC026740.1 | 0.317764 | 0.00000000004280 | postive |
| LIPT1 | AC010761.1 | 0.316044 | 0.00000000005510 | postive |
| DLAT | AP003392.4 | 0.333022 | 0.00000000000422 | postive |
| MTF1 | AC096536.2 | 0.312955 | 0.00000000008640 | postive |
| MTF1 | AL451165.2 | -0.34046 | 0.00000000000130 | negative |
| GLS | AC079921.2 | 0.303777 | 0.00000000032000 | postive |
| CDKN2A | AC009549.1 | 0.319132 | 0.00000000003490 | postive |
| GLS | AC009716.1 | 0.315949 | 0.00000000005590 | postive |
| GLS | KLF7-IT1 | 0.301475 | 0.00000000044000 | postive |
| MTF1 | SNHG16 | 0.340755 | 0.00000000000124 | postive |
| LIPT1 | AC013731.1 | 0.337462 | 0.00000000000210 | postive |
| MTF1 | GATA3-AS1 | -0.30687 | 0.00000000020700 | negative |
| LIPT1 | AC010326.3 | 0.343271 | 0.00000000000083 | postive |
| MTF1 | AC011337.1 | 0.352313 | 0.00000000000019 | postive |
| GLS | MSC-AS1 | 0.331807 | 0.00000000000510 | postive |
| LIPT1 | AC012360.3 | 0.425681 | 0.00000000000000 | postive |
| LIPT1 | AP003059.2 | 0.323177 | 0.00000000001910 | postive |
| DLD | OGFRP1 | 0.369122 | 0.00000000000001 | postive |
| MTF1 | OGFRP1 | 0.4169 | 0.00000000000000 | postive |
| LIAS | AC107068.1 | 0.360184 | 0.00000000000005 | postive |
| MTF1 | AC067852.3 | 0.30851 | 0.00000000016400 | postive |
| LIPT1 | TUG1 | 0.376544 | 0.00000000000000 | postive |
| PDHA1 | FP671120.4 | 0.448038 | 0.00000000000000 | postive |
| LIAS | STX18-AS1 | 0.380301 | 0.00000000000000 | postive |
| MTF1 | AC018653.3 | 0.308193 | 0.00000000017100 | postive |
| LIPT1 | AL590652.1 | 0.322007 | 0.00000000002280 | postive |
| CDKN2A | AL449423.1 | 0.581689 | 0.00000000000000 | postive |
| CDKN2A | CDKN2B-AS1 | 0.510309 | 0.00000000000000 | postive |
| FDX1 | AL049870.2 | 0.355199 | 0.00000000000012 | postive |
| MTF1 | AC104187.1 | -0.3053 | 0.00000000025800 | negative |
| PDHA1 | AC116407.1 | 0.355615 | 0.00000000000011 | postive |
| PDHA1 | LINC01914 | 0.333868 | 0.00000000000370 | postive |
| GLS | LINC01914 | 0.311186 | 0.00000000011200 | postive |
| LIPT1 | AC004253.1 | 0.367539 | 0.00000000000001 | postive |
| LIPT1 | OSGEPL1-AS1 | 0.310046 | 0.00000000013100 | postive |
| GLS | AL162724.2 | 0.320672 | 0.00000000002780 | postive |
| LIAS | EPB41L4A-AS1 | 0.492755 | 0.00000000000000 | postive |
| LIPT1 | LINC01004 | 0.305862 | 0.00000000023800 | postive |
| GLS | AC087286.2 | 0.317762 | 0.00000000004280 | postive |
| LIPT1 | AC011477.3 | 0.311389 | 0.00000000010800 | postive |
| LIPT1 | AC125257.1 | 0.366302 | 0.00000000000002 | postive |
| LIPT1 | PAXIP1-AS2 | 0.33196 | 0.00000000000498 | postive |
| MTF1 | AC092171.2 | 0.365376 | 0.00000000000002 | postive |
| CDKN2A | AC092171.2 | 0.335454 | 0.00000000000288 | postive |
| GLS | AL137779.1 | 0.300073 | 0.00000000053500 | postive |
| DLD | AL358394.1 | 0.391978 | 0.00000000000000 | postive |
| MTF1 | AC105001.1 | 0.367571 | 0.00000000000001 | postive |
| PDHA1 | AL138720.1 | 0.487405 | 0.00000000000000 | postive |
| PDHA1 | LINC01615 | 0.310857 | 0.00000000011700 | postive |
| PDHA1 | AC073257.2 | 0.32403 | 0.00000000001680 | postive |
| CDKN2A | AC092811.1 | 0.372431 | 0.00000000000001 | postive |
| LIAS | UBA6-AS1 | 0.341267 | 0.00000000000114 | postive |
| MTF1 | AC112491.1 | -0.34206 | 0.00000000000101 | negative |
| CDKN2A | LINC01133 | 0.317317 | 0.00000000004570 | postive |
| LIAS | ZFAS1 | 0.349951 | 0.00000000000028 | postive |
| LIAS | SNHG7 | 0.339537 | 0.00000000000151 | postive |
