# Supplementary material for: Comprehensive analysis of cuproptosis-related long noncoding RNA immune infiltration and prediction of prognosis in patients with bladder cancer
Source: Front Genet. 2022 Sep 14;13:990326. doi: 10.3389/fgene.2022.990326 (PMC9515487; doi:10.3389/fgene.2022.990326)
Supplement: Supplementary file 4 [file Table3.DOCX]

| immune | cor | *p*-value |
| --- | --- | --- |
| B cell_TIMER | -0.142131508 | 0.00475739900000 |
| T cell CD8+_TIMER | 0.301848329 | 0.00000000101000 |
| Neutrophil_TIMER | 0.277434159 | 0.00000002240000 |
| Macrophage_TIMER | 0.147977337 | 0.00327792700000 |
| Myeloid dendritic cell_TIMER | 0.367993006 | 0.00000000000005 |
| B cell plasma_CIBERSORT | -0.253853352 | 0.00000033900000 |
| T cell CD8+_CIBERSORT | -0.130521015 | 0.00958850700000 |
| T cell CD4+ naive_CIBERSORT | -0.168232413 | 0.00081294500000 |
| T cell CD4+ memory activated_CIBERSORT | 0.1774796 | 0.00040732100000 |
| T cell follicular helper_CIBERSORT | -0.21432368 | 0.00001830000000 |
| T cell regulatory (Tregs)_CIBERSORT | -0.157563523 | 0.00172900400000 |
| T cell gamma delta_CIBERSORT | 0.11386609 | 0.02397911300000 |
| Monocyte_CIBERSORT | -0.125846758 | 0.01253189500000 |
| Macrophage M0_CIBERSORT | 0.175377556 | 0.00047806000000 |
| Macrophage M1_CIBERSORT | 0.204063463 | 0.00004590000000 |
| Macrophage M2_CIBERSORT | 0.264946749 | 0.00000009760000 |
| Myeloid dendritic cell activated_CIBERSORT | -0.239699969 | 0.00000153000000 |
| Neutrophil_CIBERSORT | 0.122024944 | 0.01550360700000 |
| B cell plasma_CIBERSORT-ABS | -0.115396034 | 0.02213759700000 |
| T cell CD4+ naive_CIBERSORT-ABS | -0.169684523 | 0.00073101300000 |
| T cell CD4+ memory activated_CIBERSORT-ABS | 0.181394151 | 0.00030084100000 |
| T cell gamma delta_CIBERSORT-ABS | 0.1205072 | 0.01684522800000 |
| NK cell activated_CIBERSORT-ABS | 0.125371404 | 0.01287184100000 |
| Macrophage M0_CIBERSORT-ABS | 0.202620698 | 0.00005210000000 |
| Macrophage M1_CIBERSORT-ABS | 0.226465739 | 0.00000578000000 |
| Macrophage M2_CIBERSORT-ABS | 0.288746665 | 0.00000000552000 |
| Myeloid dendritic cell activated_CIBERSORT-ABS | -0.197431576 | 0.00008140000000 |
| Mast cell resting_CIBERSORT-ABS | 0.147562582 | 0.00336718100000 |
| Neutrophil_CIBERSORT-ABS | 0.147323019 | 0.00341973300000 |
| Macrophage M1_QUANTISEQ | 0.350306161 | 0.00000000000087 |
| Macrophage M2_QUANTISEQ | 0.109576261 | 0.02986402300000 |
| Monocyte_QUANTISEQ | 0.157772675 | 0.00170435500000 |
| Neutrophil_QUANTISEQ | -0.219776759 | 0.00001100000000 |
| NK cell_QUANTISEQ | -0.12269087 | 0.01494515400000 |
| T cell CD8+_QUANTISEQ | 0.10083944 | 0.04573929600000 |
| T cell regulatory (Tregs)_QUANTISEQ | 0.137602333 | 0.00629169800000 |
| Myeloid dendritic cell_QUANTISEQ | -0.22963711 | 0.00000424000000 |
| uncharacterized cell_QUANTISEQ | -0.118794754 | 0.01847985200000 |
| cytotoxicity score_MCPCOUNTER | 0.281837234 | 0.00000001310000 |
| NK cell_MCPCOUNTER | 0.175650697 | 0.00046826300000 |
| Monocyte_MCPCOUNTER | 0.349938846 | 0.00000000000092 |
| Macrophage/Monocyte_MCPCOUNTER | 0.349938846 | 0.00000000000092 |
| Myeloid dendritic cell_MCPCOUNTER | 0.185244188 | 0.00022194400000 |
| Neutrophil_MCPCOUNTER | -0.184230778 | 0.00024058600000 |
| Cancer associated fibroblast_MCPCOUNTER | 0.307033503 | 0.00000000050300 |
| Myeloid dendritic cell activated_XCELL | 0.305353004 | 0.00000000063100 |
| B cell_XCELL | 0.109164111 | 0.03048962500000 |
| T cell CD4+ naive_XCELL | -0.120567955 | 0.01678963300000 |
| T cell CD4+ central memory_XCELL | -0.28243293 | 0.00000001220000 |
| T cell CD8+ naive_XCELL | -0.149678803 | 0.00293381200000 |
| T cell CD8+_XCELL | -0.182178083 | 0.00028291400000 |
| T cell CD8+ central memory_XCELL | 0.105091629 | 0.03729737500000 |
| T cell CD8+ effector memory_XCELL | 0.147933972 | 0.00328715700000 |
| Myeloid dendritic cell_XCELL | 0.264119033 | 0.00000010700000 |
| Endothelial cell_XCELL | 0.13130243 | 0.00916146700000 |
| Eosinophil_XCELL | -0.214696078 | 0.00001760000000 |
| Cancer associated fibroblast_XCELL | 0.180198387 | 0.00033023700000 |
| Macrophage_XCELL | 0.385274839 | 0.00000000000000 |
| Macrophage M1_XCELL | 0.400658271 | 0.00000000000000 |
| Macrophage M2_XCELL | 0.28830482 | 0.00000000584000 |
| B cell memory_XCELL | 0.176861003 | 0.00042705500000 |
| Monocyte_XCELL | 0.323923766 | 0.00000000004730 |
| Plasmacytoid dendritic cell_XCELL | 0.259710491 | 0.00000017700000 |
| B cell plasma_XCELL | -0.103900654 | 0.03951682500000 |
| T cell CD4+ Th1_XCELL | 0.245341453 | 0.00000084900000 |
| T cell CD4+ Th2_XCELL | 0.362200286 | 0.00000000000013 |
| immune score_XCELL | 0.235415796 | 0.00000238000000 |
| stroma score_XCELL | 0.176107027 | 0.00045231000000 |
| microenvironment score_XCELL | 0.237392104 | 0.00000194000000 |
| Cancer associated fibroblast_EPIC | 0.259471831 | 0.00000018200000 |
| T cell CD4+_EPIC | -0.32734164 | 0.00000000002880 |
| Macrophage_EPIC | 0.305785818 | 0.00000000059600 |
| NK cell_EPIC | 0.336519268 | 0.00000000000734 |
| uncharacterized cell_EPIC | -0.102825637 | 0.04161509400000 |
